# Different Accumulation of Free Amino Acids during Short- and Long-Term Osmotic Stress in Wheat

**DOI:** 10.1100/2012/216521

**Published:** 2012-08-01

**Authors:** Zita Kovács, Livia Simon-Sarkadi, Ildikó Vashegyi, Gábor Kocsy

**Affiliations:** ^1^Department of Applied Biotechnology and Food Science, Budapest University of Technology and Economics, P.O. Box 91, 1521 Budapest, Hungary; ^2^Agricultural Institute, Centre for Agricultural Research, Hungarian Academy of Sciences, P.O. Box 19, 2462 Martonvásár, Hungary; ^3^Research Institute of Chemical and Process Engineering, Faculty of Information Technology, University of Pannonia, P.O. Box 158, 8200 Veszprém, Hungary

## Abstract

The effect of wheat chromosome 5A on free amino acid accumulation induced by osmotic stress was compared in chromosome 5A substitution lines with different freezing tolerance. Treatment with 15% polyethylene glycol (PEG) resulted in greater total free amino acid content even after 3 days compared to the controls. The ratio of amino acids belonging to various amino acid families differed after 3-week treatment in the control and PEG-treated plants only in the case of the freezing-sensitive substitution line. There was a transient increase with a maximum after 3 days in the amounts of several amino acids, after which their concentrations exhibited a more gradual increase. During the first days of osmotic stress the Glu, Gln, Asp, Asn, Thr, Ser, Leu, and His concentrations were greater in the tolerant substitution line than in the sensitive one, while the opposite relationship was observed at the end of the PEG treatment. The coordinated changes in the levels of individual amino acids indicated that they are involved in both the short- and long-term responses to osmotic stress. The alterations differed in the two chromosome 5A substitution lines, depending on the stress tolerance of the chromosome donor genotype.

## 1. Introduction

Abiotic stresses result in severe injury and yield loss in crops. A common consequence of drought and low temperature is the induction of osmotic stress. The accumulation of compatible solutes, such as certain amino acids especially Pro, prevents the development of severe osmotic stress. Amino acids also have several other roles in plants, for example they regulate ion transport and stomatal opening and affect the synthesis and activity of enzymes, gene expression, and redox homeostasis, helping the plants to cope with the harmful effects of osmotic stress [1].

Osmotic stress, which can be induced by limited water supply or osmotic agents, alters both the amino acid pattern and the concentrations of individual amino acids. Osmotic stress induced by polyethylene glycol resulted in an increase in the concentrations of Asp, Glu, Asn, Thr, Ser, Ala, and Pro during the first day of treatment in the roots of maize [2]. After dehydration the amino acid contents changed to differing extents in the desiccation-tolerant young leaves and sensitive old leaves of the resurrection plant *Sporobolus stapfianus* [3], indicating that these changes depended on the level of stress tolerance.

Proline plays a very important role as an osmoprotectant in the adaptation to osmotic stress [4]. The accumulation induced by osmotic stress is based on increased synthesis and decreased degradation [5]. Osmotic stress resulted in a greater increase in Pro content in a drought-tolerant rice genotype than in a sensitive one, due to the greater expression of the gene encoding pyrroline-5-carboxylate synthase and activity of this enzyme involved in the Pro synthesis [6]. Further evidence for the protective role of Pro was found in transgenic plants, where its overproduction increased tolerance to osmotic stress [5, 7]. 

Besides Pro, the involvement of other amino acids in the response to osmotic stress was also demonstrated. Arginine was found to function as a compatible solute, improving stress tolerance in yeast [8]. Under hyperosmotic conditions the transcription of genes encoding the enzymes involved in Arg biosynthesis increased, while those involved in degradation decreased. Both exogenous Arg and the overproduction of two enzymes required for its synthesis increased tolerance. In addition, osmotic stress resulted in the increased expression of asparagine synthase genes in sunflower [9] and in wheat [10]. The overexpression of glutamine synthase increased tolerance to osmotic stress in rice [11]. These observations show that the changes in amino acid concentrations induced by osmotic stress may be due to the altered expression of genes encoding the enzymes involved in their metabolism. 

Chromosome 5A of wheat was found to affect the cold-induced accumulation of certain free amino acids [12]. Since this chromosome has an important role in the stress response and controls the expression of several stress-related genes [13], it seemed likely that it was involved in controlling the effect of osmotic stress on free amino acid levels. This hypothesis was tested by comparing the changes induced by short- and long-term osmotic stress in the free amino acid composition and in the concentration of individual amino acids in wheat chromosome 5A substitution lines with different freezing tolerance. Osmotic stress also occurs during freezing, so these genotypes were expected to give different responses.

## 2. Materials and Methods

### 2.1. Plant Material and Treatment

A specific genetic system consisting of the moderately freezing-sensitive wheat (*Triticum aestivum *L. ssp.* aestivum*) cv. Chinese Spring (CS) and two chromosome 5A substitution lines the freezing-tolerant Chinese Spring (Cheyenne 5A) (CS(Ch5A)) and the freezing-sensitive Chinese Spring (*Triticum aestivum* L. ssp.* spelta* 5A) (CS(Tsp5A)) was used in the experiments [14]. The seeds were obtained from the Martonvásár Cereal Gene Bank (Agricultural Institute, Agricultural Research Centre, Hungarian Academy of Sciences, Martonvásár, Hungary). According to previous studies in which the osmotic stress tolerance of the used genotypes was also checked, CS and CS(Tsp5A) are sensitive and CS(Ch5A) is tolerant [15, 16]. It was assumed thus difference in the tolerance may also affect the accumulation of amino acids during osmotic stress.

Seedlings were grown in hydroponic culture using half-strength modified Hoagland nutrient solution [17] in a plant growth chamber (Conviron PGV-36, Controlled Environments Ltd., Canada) at 18/15°C day/night temperature and 70/75% relative humidity with 16 h illumination at 270 *μ*moL photons m^−2^ s^−1^ for 12 days prior to the stress period. Treatment with 15% polyethylene glycol (PEG) lasted for 21 days, under cultivation conditions similar to those described above. Control plants were cultivated without any supplementation. Samples were taken at the beginning of the treatment and after 1, 3, 7, and 21 days. The experiments were repeated three times and in each experiment three parallels were investigated. 

### 2.2. Determination of Free Amino Acid Content

Shoot samples of 300–600 mg fresh weight were crushed in liquid nitrogen and extracted with 2 mL cold 10% trichloroacetic acid for 1 hour with gentle agitation on a shaker (C. Gerhardt GmbH & Co. KG, Germany) at room temperature. Each sample was filtered through a 0.2 *μ*m pore membrane filter (Sartorius AG, Germany). The biochemical analysis was carried out on an automatic amino acid analyser (Ingos Ltd., Czech Republic) equipped with an Ionex Ostion LCP5020 cation-exchange column (22 × 0.37 cm). Free amino acids were separated by stepwise gradient elution using a Li^+^-citric buffer system (Ingos Ltd., Czech Republic). Colorimetric detection was accomplished at 570 nm and 440 nm (for Pro) after postcolumn derivatisation with ninhydrin reagent. 

### 2.3. Statistical Analysis

The free amino acid data were evaluated using analysis of variance and the significant differences were calculated. 

## 3. Results

### 3.1. Effect of Osmotic Stress on Plant Growth

**Figure 1:**
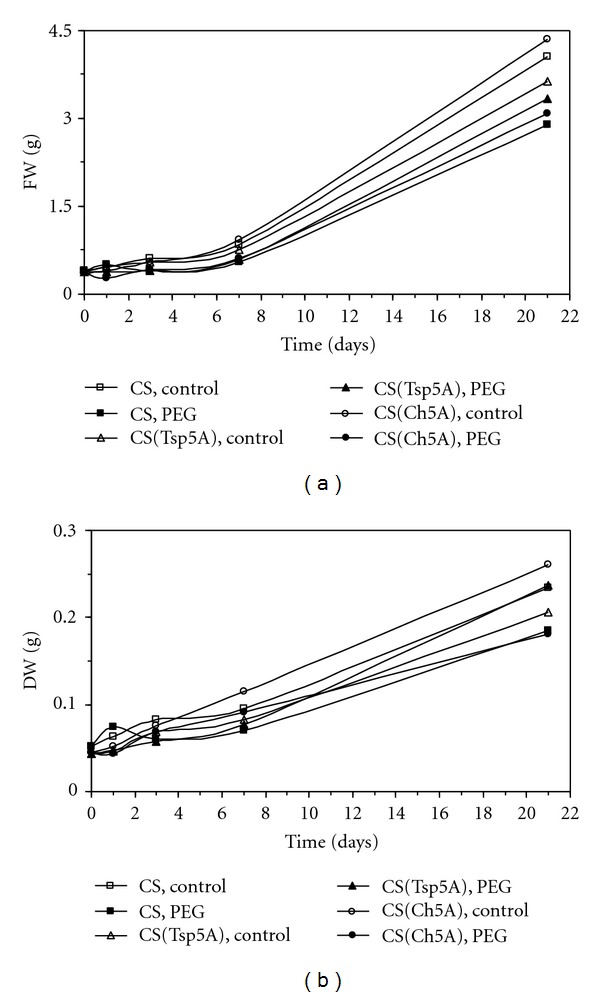
Effect of osmotic stress on fresh ((a); significant difference (SD): 0.612) and dry weight ((b); SD: 0.065). The time course of changes was compared under control and stress (15% PEG) conditions in the freezing-sensitive CS and CS(Tsp5A) and the freezing-tolerant CS(Ch5A) wheat genotypes. The standard deviations of the data were below 10% in each case (not shown on the graphs because of the better transparency).

Application of PEG in 15% concentration resulted only in mild osmotic stress as it was shown by growth data (Figure 1). There were only very slight changes in fresh (Figure 1(a)) and dry (Figure 1(b)) weight of the shoots during the first week of the treatment. The growth of the plants sped up during the subsequent two weeks. The fresh and dry weight data did not differ significantly between the treated and control plants and the three genotypes at the end of the osmotic stress. The plants had no visible injuries at the end of the 3-week osmotic stress. 

### 3.2. Effect of Osmotic Stress on Plant Amino Acid Composition

**Figure 2:**
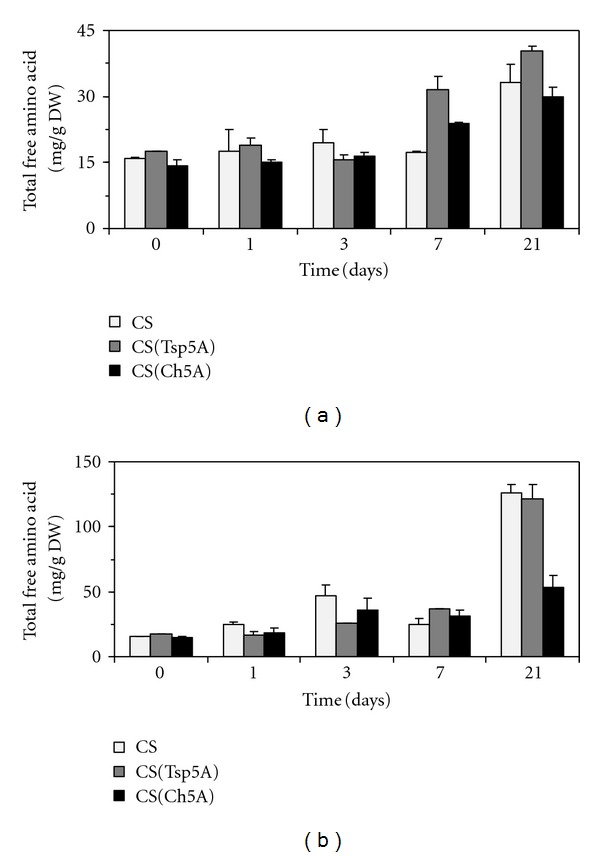
Effect of osmotic stress on total free amino acid content. The time course of changes was compared under control ((a); SD: 4.47) and stress (15% PEG; (b); SD: 12.68) conditions in the freezing-sensitive CS and CS(Tsp5A) and the freezing-tolerant CS(Ch5A) wheat genotypes.

**Figure 3:**
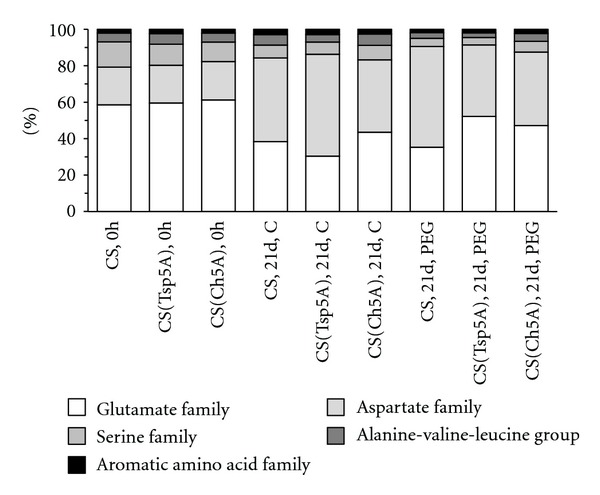
Changes in the ratio of amino acid families during osmotic stress. A comparison was made of the freezing-sensitive CS and CS(Tsp5A) and the freezing-tolerant CS(Ch5A) wheat genotypes, grown for three weeks under control (C) or stress conditions (15% PEG). The experiment was repeated with three parallels three times with similar results.

The total free amino acid concentration increased already after 3 days osmotic stress in all the genotypes, but a great difference between control and treated plants was observed after 3 weeks, especially in the case of CS and CS(Tsp5A) (Figure 2). Comparing the two substitution lines following PEG treatment, the total free amino acid content was relatively greater after 3 d in the freezing-tolerant CS(Ch5A) and after 21 d in the freezing-sensitive CS(Tsp5A). The ratio of amino acids belonging to the various amino acid families changed similarly during the 3-week treatment in the control and PEG-treated plants except for the greater difference in the ratio of glutamate and aspartate family in CS(Tsp5A) (Figure 3). 

**Figure 4:**
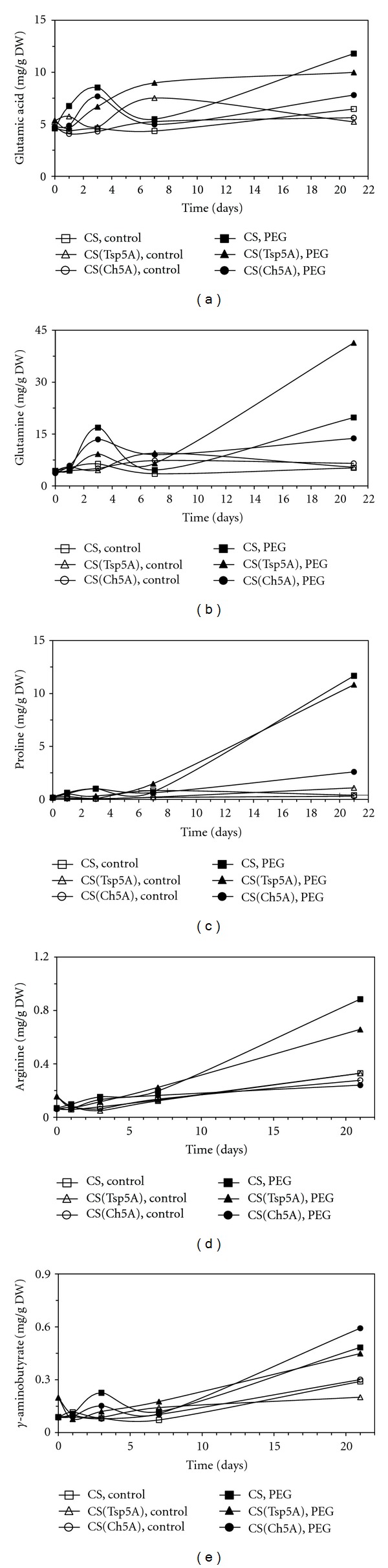
Effect of osmotic stress on the concentration of amino acids belonging to the glutamate family. The time course of changes was compared under control and stress (15% PEG) conditions in the freezing-sensitive CS and CS(Tsp5A) and the freezing-tolerant CS(Ch5A) wheat genotypes. The standard deviations of the data were below 10% in each case (not shown on the graphs because of the better transparency). (a): Glu (SD: 1.66), (b): Gln (SD: 5.10), (c): Pro (SD: 1.67), (d): Arg (SD: 0.070), and (e): *γ*-aminobutyrate (SD: 0.128).

**Figure 5:**
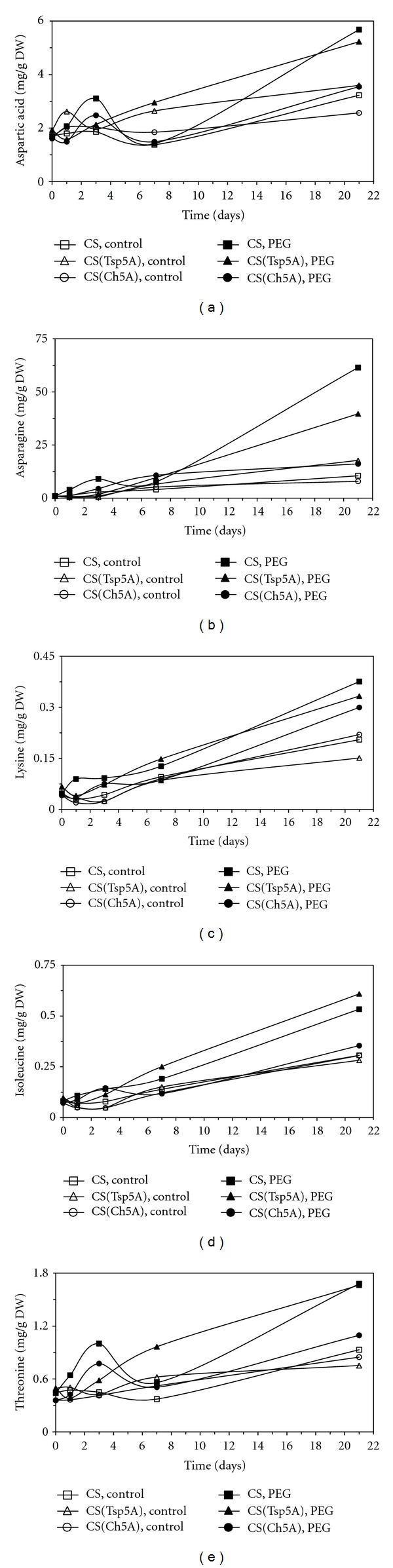
Effect of osmotic stress on the concentration of amino acids belonging to the aspartate family. The time course of changes was compared under control and stress (15% PEG) conditions in the freezing-sensitive CS and CS(Tsp5A) and the freezing-tolerant CS(Ch5A) wheat genotypes. The standard deviations of the data were below 10% in each case (not shown on the graphs because of the better transparency). (a): Asp (SD: 0.505), (b): Asn (SD: 5.92), (c): Lys (SD: 0.037), (d): Ile (SD: 0.050), and (e): Thr (SD: 0.204).

**Figure 6:**
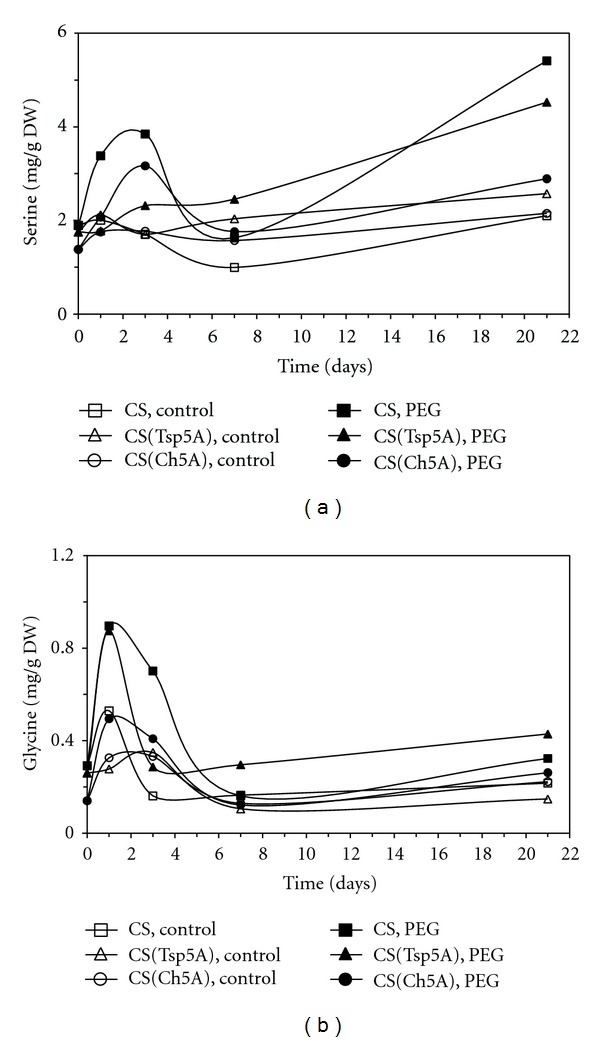
Effect of osmotic stress on the concentration of amino acids belonging to the serine family. The time course of changes was compared under control and stress (15% PEG) conditions in the freezing-sensitive CS and CS(Tsp5A) and the freezing-tolerant CS(Ch5A) wheat genotypes. The standard deviations of the data were below 10% in each case (not shown on the graphs because of the better transparency). (a): Ser (SD: 0.644), and (b): Gly (SD: 0.159).

**Figure 7:**
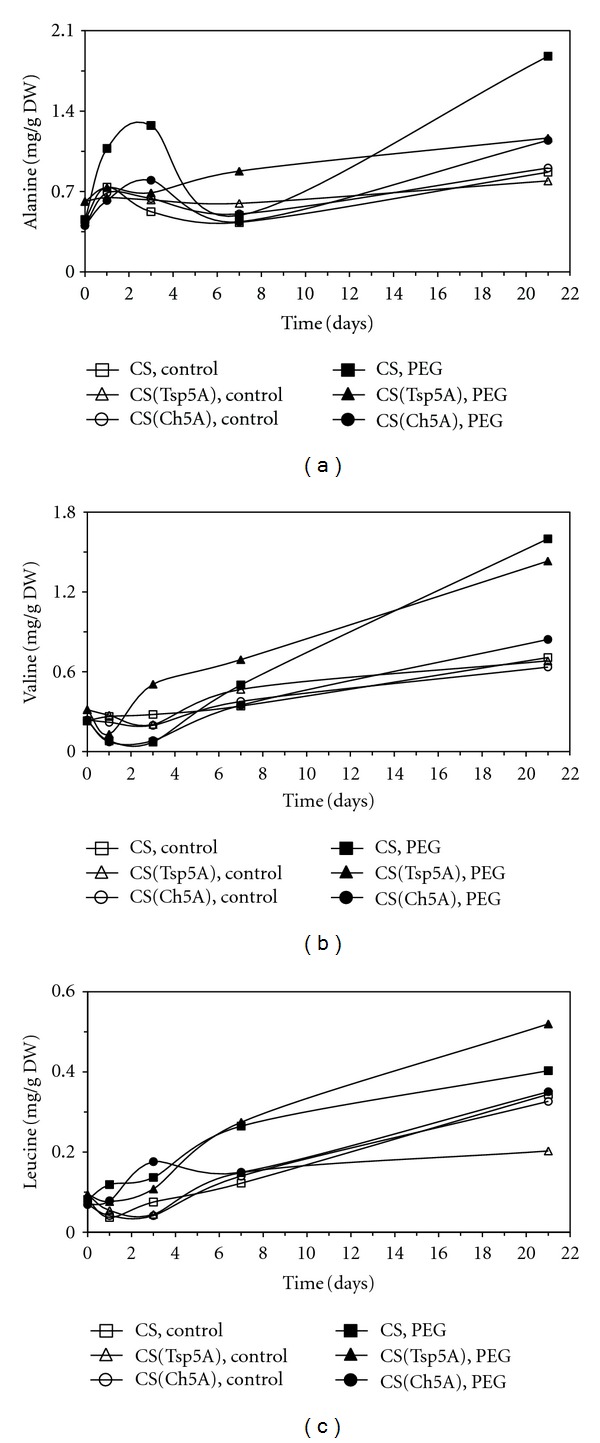
Effect of osmotic stress on the concentration of amino acids belonging to the pyruvate family. The time course of changes was compared under control and stress (15% PEG) conditions in the freezing-sensitive CS and CS(Tsp5A) and the freezing-tolerant CS(Ch5A) wheat genotypes. The standard deviations of the data were below 10% in each case (not shown on the graphs because of the better transparency). (a): Ala (SD: 0.261), (b): Val (SD: 0.146), and (c): Leu (SD: 0.058).

**Figure 8:**
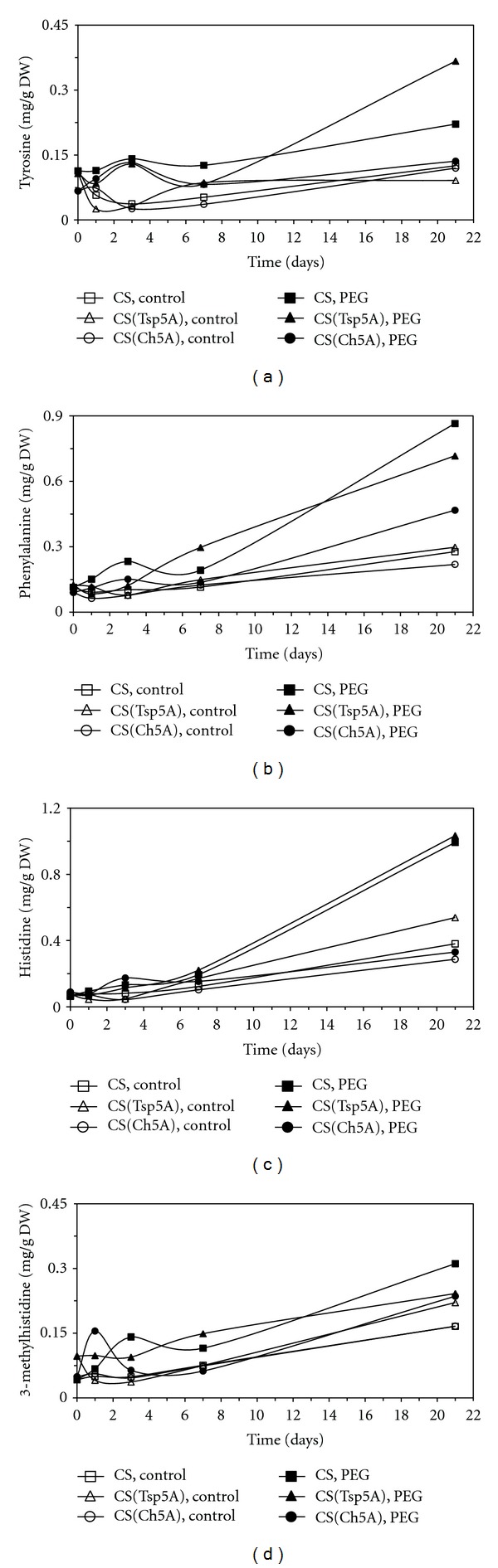
Effect of osmotic stress on the concentration of amino acids belonging to the aromatic amino acid family. The time course of changes was compared under control and stress (15% PEG) conditions in the freezing-sensitive CS and CS(Tsp5A) and the freezing-tolerant CS(Ch5A) wheat genotypes. The standard deviations of the data were below 10% in each case (not shown on the graphs because of the better transparency). (a): Tyr (SD: 0.046), (b): Phe (SD: 0.091), (c): His (SD: 0.122), and (d): 3-metil-His (SD: 0.026).

In the case of the individual amino acids little or no change was observed during the 3 weeks of treatment in the control plants, while the level of most of the 19 amino acids examined was increased by osmotic stress (Figures 4–8). Their concentrations were significantly greater in the PEG-treated plants than in control ones at the most sampling points. The levels of several amino acids were relatively greater after 3 d of PEG treatment in CS(Ch5A) and after 3 weeks in CS(Tsp5A). 

### 3.3. Changes of Glutamate Family during Osmotic Stress

The glutamate content exhibited a very rapid PEG-induced increase during the first three days of treatment, but later its concentration transiently decreased, then increased again in CS and CS(Ch5A) (Figure 4(a)). The changes in the glutamine content were very similar (Figure 4(b)). There was a peak in its concentration after 3 days and after a transient reduction its level increased again during the second half of the treatment. Although the Pro concentration exhibited a great increase (more than 10-fold) in all genotypes, its maximum level was 3.3-fold greater in CS(Tsp5A) than in CS(Ch5A) (Figure 4(c)). Slight changes occurred only in the Arg content during the first week of the treatment in all 3 genotypes, but later on it increased in CS and CS(Tsp5A) to high levels (Figure 4(d)). The gamma-aminobutyrate content exhibited first a transient decrease in CS(Tsp5A), and a transient increase in CS and CS(Ch5A) which was followed by a similar increase in all 3 genotypes (Figure 4(e)). The amounts of Glu and Gln were greater after 3 d in CS(Ch5A) and CS than in CS(Tsp5A), while after 21 d osmotic treatment higher levels of these amino acids were detected in CS(Tsp5A) and CS than in CS(Ch5A).

### 3.4. Changes of Aspartate Family during Osmotic Stress

The aspartate concentration exhibited a transient increase during the first days of the experiment with a local maximum after 3 d of treatment in CS(Ch5A) and CS (Figure 5(a)). Later a second increase was observed in these two genotypes, and the level gradually increased in CS(Tsp5A). Similarly to Asp, the greatest asparagine concentrations were detected after 3 weeks of osmotic stress in CS(Tsp5A) and CS (Figure 5(b)). A transient increase was found in the amount of this amino acid during the first week of the treatment in CS, while its concentration gradually increased in CS(Tsp5A). The Lys content increased gradually to similar level in all 3 genotypes after PEG treatment (Figure 5(c)). The Ile content reached higher concentrations after 3 weeks of osmotic stress in CS and CS(Tsp5A) than in CS(Ch5A) (Figure 5(d)). The Thr concentration exhibited a local maximum after 3 d in CS and CS(Ch5A), while it gradually increased throughout the experiment in CS(Tsp5A) (Figure 5(e)). The concentration of Asp and Thr was greater in CS(Ch5A) and CS than in CS(Tsp5A) after 3 d osmotic stress, and the level of Asp, Asn, Ile, and Thr was greater in CS(Tsp5A) and CS than in CS(Ch5A) after 21 d.

### 3.5. Changes of Other Amino Acid Families during Osmotic Stress

Two members of serine amino acid family could be detected. The Ser concentration exhibited a large transient increase during the first week of the treatment in CS and CS(Ch5A), while it gradually increased in CS(Tsp5A) during the 3-week osmotic stress (Figure 6(a)). The Ser content increased during the second half of the treatment again in CS. The Gly content reached a maximum after 1 d osmotic stress, then had a rapid decrease, and it was low after 1 week PEG-treatment. (Figure 6(b)).

All 3 members of the pyruvate family were studied. The Ala concentration showed greater changes only in CS, where it had a transient increase during the first week, and increased again during the second half of the experiment (Figure 7(a)). The Val concentration first decreased in all the genotypes, but subsequently it increased and this change was quicker in CS and CS(Tsp5A) than in CS(Ch5A) (Figure 7(b)). The Leu content increased in all three genotypes during the whole stress period (Figure 7(c)).

Four aromatic amino acids could be measured. The Tyr concentration, after an initial transient increase, exhibited a rapid increase in CS(Tsp5A) and a slow one in the other two genotypes (Figure 8(a)). The Phe content, except for a small transient decrease in CS and CS(Ch5A) after 3 days of PEG treatment, was increased by osmotic stress, and this change was much greater in CS and CS(Tsp5A) than in the other genotypes (Figure 8(b)). The time course of the alterations in His content was similar to that of Phe content (Figure 8(c)). The concentration of 3-methyl-His increased quickly during the first days of PEG treatment in CS and CS(Ch5A), which was followed by a decrease and subsequently by a slow increase (Figure 8(d)).

## 4. Discussion

Interestingly, the fresh and dry weight data of control and treated plants did not differ at the end of the 3-week treatment with 15% PEG in the present experiment, however on the basis of shoot damage monitored after two weeks recovery phase following the 3-week osmotic stress in a previous study, the 3 genotypes had different level of tolerance [15, 16]. These controversial results can be observed by the phenomenon that the stress injuries appear very often not during the stress, but during the subsequent recovery phase. While during longer osmotic stress (7 and 21 d) the wheat variety Ch and the derived CS(Ch5A) chromosome substitution line seemed to be more tolerant to osmotic stress than the genotypes CS, Tsp and CS(Tsp5A) [15, 16], after short, 1-day exposure to osmotic stress only Ch proved to be more tolerant than the other genotypes [18]. Since dry weight data exhibited a smaller (5-fold) change than fresh weight data (10-fold) during the 3-week osmotic stress, the former one is more appropriate for usage as basis of the amino acid concentrations. In addition, the difference between control and treated plants was smaller in dry weight than in fresh weight at the end of the experiment. 

Despite the great changes induced by osmotic stress in the amounts of most amino acids, the ratio of the amino acid families was similar after 3 weeks in the control and PEG-treated plants except for CS(Tsp5A), indicating that the alterations observed were coordinated. A similar coordination was found in the level of minor amino acids in the leaves of wheat and potato grown under various photosynthetic conditions [19]. In addition, a coordinated increase in the concentration of amino acids derived from pyruvate and oxalacetate was observed during cold shock in *Arabidopsis* [20]. The genetic modification of the Pro level affected the concentrations of several other amino acids in soybean [21]. These observations may be explained by the cross-pathway regulation of key enzymes of biosynthesis, but the involvement of protein degradation and storage cannot be excluded.

In the case of many amino acids (Glu, Gln, Asp, Thr, Ser, Gly, Ala, Tyr) a rapid transient increase was first observed with a local maximum after 3 days of PEG treatment, after which their levels increased again from the 7th day of osmotic stress. These results suggest that an elevation in the amino acid content is involved in both the short- and long-term response to osmotic stress. The initial increase was 1.5-2-fold greater in the case of several amino acids in the freezing-tolerant CS(Ch5A) than in the freezing-sensitive CS(Tsp5A) after osmotic stress (present study) and cold [12], indicating that the early accumulation of free amino acids contributes to a greater level of stress tolerance. After 3 weeks of stress other protective mechanisms may become more important, since the amino acid content was greater in the sensitive CS(Tsp5A). Thus, the level of the antioxidants ascorbate and glutathione (GSH) was greater after 3 weeks of cold in CS(Ch5A) than in CS(Tsp5A) [22]. The increase in GSH concentration may be due to the early stress-induced accumulation of Glu, which is a precursor of GSH.

The special role of Pro in the stress response, described in several publications [5, 23], was corroborated in the present study. The increase in the Pro content may originate from the induction of pyrroline 5-carboxylate synthetase, described at both the gene expression and activity levels in rice subjected to drought [6]. Besides its osmoprotective role, Pro may also function as an antioxidant. The stress-induced increase in its synthesis could be important for the maintenance of a low NADPH : NADP^+^ ratio and could prevent the accumulation of NADPH, which occurs due to the inhibition of the Calvin cycle under stress conditions [23]. The recycling of NADP^+^ is important as it provides on electron acceptor for photosynthesis, thus preventing the accumulation of reactive oxygen species.

In contrast to several other amino acids, there was no further great increase in the Glu content after the initial transient rise. This can be explained by its intensive use for the synthesis of Pro, polyamines, and GSH during the long-term (3-week) stress response. This assumption is confirmed by the observation that the spermine content increased in wheat after 3 weeks of osmotic stress [16]. As in the case of the Glu content in wheat, a relationship was found between the activity of enzymes involved in Glu synthesis and stress tolerance in the leaves of the resurrection plant *Sporobolus stapfianus* [3]. The high demand for Glu under stress conditions due to its use for the synthesis of Pro, polyamines, and GSH can be met by the conversion of Gln to Glu, since Gln accumulation was also induced by osmotic stress. 

The protective role of Arg during osmotic stress, indicated by the fact that its level changed to different extents in wheat genotypes with different tolerance, may be due to its osmoprotective function, observed in yeast [8], or to its use for polyamine synthesis. Positively charged polyamines are involved in the stress response through their interaction with negatively charged macromolecules, such as DNA, RNA, proteins, and phospholipids, resulting in changes in the physical and chemical properties of the membranes, in the structure of nucleic acids and in enzyme activities [24].

The comparison of two chromosome 5A substitution lines with different levels of stress tolerance revealed that this chromosome affects both the short- and long-term changes in the free amino acid concentration. This effect is probably based on the regulatory role of the Cbf/Dreb (C-repeat binding/drought-responsive element binding) transcription factors mapped at the *Fr2* (frost-resistance 2) locus on the long arm of chromosome 5A in the response to drought and frost [25]. *Cbf*s localised on chromosome 5A affect the expression of genes involved in the amino acid metabolism, as shown by a comparison of cold-induced changes in the transcriptome of the Cs(Tsp5A) and Cs(Ch5A) substitution lines [13]. Like those of amino acids, the levels of polyamines were also affected by chromosome 5A during osmotic stress in wheat [26]. Although in the present study we focused on chromosome 5A because of its importance in the stress response [13], the influence of other chromosomes on osmotic stress-induced amino acid accumulation can not be excluded, since several chromosomes were shown to affect osmoregulation in wheat [27]. Thus, chromosome 5D had greater effect on PEG-induced carbohydrate accumulation than chromosome 5A [28].

In summary, coordinated changes in the levels of individual amino acids indicate that they are involved in both the short- and long-term response to osmotic stress. The alterations differed for the two chromosomes 5A substitution lines, depending on the stress tolerance of the chromosome donor genotype.
